# O-GlcNAcylation and immune cell signaling: A review of known and a preview of unknown

**DOI:** 10.1016/j.jbc.2024.107349

**Published:** 2024-05-06

**Authors:** Parameswaran Ramakrishnan

**Affiliations:** 1 Department of Pathology, Case Western Reserve University, Cleveland, Ohio, USA; 2 The Case Comprehensive Cancer Center, Case Western Reserve University, Cleveland, Ohio, USA; 3 Department of Biochemistry, Case Western Reserve University, Cleveland, Ohio, USA; 4 University Hospitals-Cleveland Medical Center, School of Medicine, Case Western Reserve University, Cleveland, Ohio, USA

**Keywords:** O-GlcNAcylation, hexosamine biosynthetic pathway, posttranslational modifications, immune system, cell signaling, immunometabolism, hyperglycemia, diabetes, inflammation, autoimmunity, cancer

## Abstract

The dynamic and reversible modification of nuclear and cytoplasmic proteins by O-GlcNAcylation significantly impacts the function and dysfunction of the immune system. O-GlcNAcylation plays crucial roles under both physiological and pathological conditions in the biochemical regulation of all immune cell functions. Three and a half decades of knowledge acquired in this field is merely sufficient to perceive that what we know is just the prelude. This review attempts to mark out the known regulatory roles of O-GlcNAcylation in key signal transduction pathways and specific protein functions in the immune system and adumbrate ensuing questions toward the unknown functions.

## Overview of O-GlcNAcylation in the immune system

O-GlcNAcylation is a posttranslational modification (PTM) of serine and threonine residues of cytoplasmic and nuclear proteins with the sugar GlcNAc. It is a dynamic and reversible process, the cycling of which is regulated by two enzymes: O-GlcNAc transferase (OGT) and O-GlcNAcase (OGA) (1). OGT and OGA are ubiquitously expressed and the genetic knockout of OGT in a mouse model shows embryonic lethality (2), while that of OGA shows perinatal lethality (3), underscoring the vital role of O-GlcNAcylation in survival. The substrate UDP-GlcNAc for O-GlcNAcylation is generated as part of the glucose metabolism through the hexosamine biosynthetic pathway (HBP) (4, 5). O-GlcNAcylation controls protein functions in physiological and pathological conditions, including the regulation of homeostatic immune cell functions, inflammation, autoimmunity, and the response to infections (6, 7). High expression levels of OGT and OGA have been shown to occur in immune cells (8), which suggest potential rapid cycling of O-GlcNAcylation regulating protein activity in the immune system. O-GlcNAcylation may affect protein function by altering structure, stability, and localization, all of which will influence protein–protein and protein–nucleic acid interactions. O-GlcNAcylation also positively and negatively influences other PTMs, including phosphorylation, methylation, acetylation, and ubiquitination (4, 9, 10). This review attempts to discuss these O-GlcNAcylation–dependent biochemical events involved in immune cell signaling, reiterate the known roles of O-GlcNAcylation, tries to derive knowledge relevant to the immune cells from its role in nonimmune cells and raises some plausible questions with potential to fill knowledge gaps in the field.

## O-GlcNAcylation in immune system development

Global knockouts of OGT (2) and OGA (3) yield lethal phenotypes, suggesting the critical role of the addition and removal of O-GlcNAcylation in development. Consistent with this, the conditional knockout of OGT in hematopoietic stem cells (HSCs) showed the essential role of OGT in the maintenance of HSCs and hematopoiesis, as indicated by a decrease in both lymphoid and myeloid compartments in mice (11). Similarly, the conditional deletion of OGA in HSCs decreased early thymic T cell development and peripheral lymphoid cells, indicating the need for O-GlcNAc cycling (12). Furthermore, the conditional deletion of OGT in thymocytes using the Lck-Cre transgene, demonstrated its essential role in all stages of thymic T cell development, with its most significant effect on CD4^+^ T cell development (13). O-GlcNAcylation not just positively regulates T cell development; it seems to possess a negative regulatory role as well. Metabolic studies on early T cell development showed that the transition from a double negative stage to a double positive stage of T cell development is associated with a significant decrease in O-GlcNAcylation; however, further development of double positive T cells was associated with an increase in O-GlcNAcylation, suggesting a developmental stage-dependent function of O-GlcNAcylation (14). The key signaling proteins regulating T cell development such as NOTCH, c-Myc, NF-κB, and nuclear factor of activated T cells (NFAT) are all O-GlcNAcylated; however, comprehensive mechanistic studies demonstrating the biochemical roles of the O-GlcNAcylated versions of these proteins in T cell development are lacking (14). The specific deletion of OGT in T regulatory cells (Treg) showed that thymic Treg cell development does not depend on OGT; however, effector Treg cell development and its suppressive function were dependent on OGT, the O-GlcNAcylation of forkhead box P3 (FOXP3) and signal transducer and activator of transcription 5 (STAT5), and interleukin-2 (IL-2) signaling (15).

In contrast to that of T cells, knowledge on the role of O-GlcNAcylation in other immune cells remains poorly defined. The conditional knockout of OGT in B cells using CD19-Cre did not affect any stages of B cell development; however, it decreased the number of mature B cells that was attributed to increased apoptosis (16). But, the conditional deletion of OGT in bone marrow stromal cells prevented the transition from pre-pro-B to pro-B cells, with a loss of mature B cells in the periphery, with no effect on T cells, suggesting that O-GlcNAcylation in stromal cells is crucial to provide the microenvironment necessary for B cell development (17). Interestingly, the same study showed that OGT deficiency in bone marrow stromal cells results in an increase in myeloid cell development possibly through the activation of CCAAT/enhancer binding protein beta and stem cell factor expression as well as an increase in neutrophils (17). The role of O-GlcNAcylation in myeloid cell differentiation has also been shown in a study where OGA inhibition in normal hematopoietic progenitor cells promoted its differentiation to dendritic cells (DCs) (18). Likewise, OGT inhibition leads to the blockage of DC development from human monocytes possibly through modulating mitogen activated protein kinase phosphorylation (19). Knowledge on the exact role of O-GlcNAcylation on macrophage development is minimal. Studies using monocytic cell lines of acute myeloid leukemia origin show that OGT inhibition results in a differentiation tendency toward macrophage lineage (18). No countable studies exist for the role of O-GlcNAcylation in the development of other immune cells, such as basophils, eosinophils, mast cells, and innate lymphoid cells (Table 1). Further comprehensive studies on HSC, each immune cell type–specific OGT and OGA conditional KO mice, or transgenic mice overexpressing OGT or OGA are expected to yield further knowledge on the developmental roles of O-GlcNAcylation in the immune system.

**Table 1:** Unknowns of O-GlcNAcylation–dependent signaling in innate immune cell functions

Neutrophil signaling	Myeloid signaling	NK cell signaling	Mast cell signaling
• NET formation • Neutrophilia • Antimicrobial functions in normal and hyperglycemic conditions • Dynamics of first line defense • Chemotaxis and motility in the absence of chemotactic factor • Diabetic wound healing	• Monocyte differentiation • Antigen presentation • Role in monocytes, macrophages and DC in viral and bacterial infections • Tissue-specific regulation of myeloid cells • Roles in cDC, pDC, moDC • O-GlcNAc proteome in myeloid cells	• Differential regulation of O-GlcNAcylation by cytokines and target cells • Identification of O-GlcNAcylated proteins controlling cytotoxicity • Mechanisms controlling detection of virus infected and tumor cells • Role in tissue resident and circulating NK cells	• Degranulation in allergy • Wound healing • Hyperglycemia-induced changes in mast cell functions • Physiological roles such as vasodilation and angiogenesis • Mast cells in gut–brain axis • Mast cell activation syndrome and mastocytosis

Other unknown roles of O-GlcNAcylation in the immune system			

• Role in innate immune cells such as basophils, eosinophils, and innate lymphoid cells as well as in NKT cells with overlapping functions in the innate and adaptive immune system • Role in the stromal cells of lymphoid organs that support immune cell development and maintenance • Role in immunodeficiency resulting in partial or complete impairment of the immune response			

Representation of major functions of the selected innate immune cell types that needs further studies to validate or refute the involvement of O-GlcNAcylation.

Abbreviations: cDC, conventional dendritic cell; DC, dendritic cell; moDC, monocyte-derived dendritic cell; NET, neutrophil extracellular trap; NK, natural killer; pDC, plasmacytoid dendritic cell.

## Biochemical regulation of innate immune signaling by O-GlcNAcylation

Innate immune responses trigger most rapid cellular signaling mechanisms in immune cells such as neutrophils, monocytes, macrophages, DCs, natural killer (NK) cells, basophils, eosinophils, mast cells, and innate lymphoid cells. O-GlcNAcylation has been shown to regulate several of these innate immune cell functions by modulating transcription factor activity, signaling pathways, and protein–protein interactions.

## O-GlcNAcylation in neutrophil signaling

Neutrophils are the first responders in the innate immune system and constitute the majority of white blood cells. Early studies have shown that formylated Met-Leu-Phe acts as a potent chemoattractant for neutrophils by recruiting them to the infection site (20). In an elegant biochemical study, Kneass and Marchase showed that neutrophil stimulation with formylated Met-Leu-Phe or exogenous glucosamine results in enhanced O-GlcNAcylation and glucosamine specifically promoted chemotaxis without affecting the production of reactive oxygen species by neutrophils (21). Interestingly, this study also showed that the enhancement of O-GlcNAcylation augments basal chemotactic activity in the absence of a chemotactic factor, suggesting a role for O-GlcNAcylation in neutrophil motility. In a sequel study, the same group showed that enhanced O-GlcNAcylation activates extracellular signal–regulated kinase (ERK) pathways in neutrophils controlling chemotaxis (22). It was also shown that O-GlcNAcylation rapidly increases within 2 min of activation in neutrophils, suggesting its key role in signal transduction (23). In spite of these strong foundational evidences and preliminary indications on the O-GlcNAcylation of Rac (24), p38, and ERK (25), no in depth studies exist on identifying O-GlcNAcylated proteins and their mechanistic roles in regulating neutrophil activation and function.

The role of neutrophil O-GlcNAcylation remains largely unknown and it is of prime interest to know how neutrophil function changes in diabetes, where global O-GlcNAcylation may remain elevated depending on the hyperglycemic state. Neutrophils play a key role in chronic diabetic inflammation and ulcers and hyperglycemia has been shown to promote the formation of neutrophil extracellular traps that delay wound healing and contribute to other secondary complications in diabetes (26). Whether O-GlcNAcylation plays a role in these hyperglycemia-associated pathologies needs to be addressed. In addition, how O-GlcNAcylation regulates the antimicrobial actions of neutrophils in healthy individuals with normoglycemia floats as a lingering question, the answer to which will significantly deepen our knowledge on the mechanisms involved in neutrophil activation and function (Table 1).

## O-GlcNAcylation in myeloid cell signaling

Monocyte and macrophage actions follow neutrophils in terms of the speed of innate immune responses. Monocytes are relatively short-lived cells in circulation before they die or differentiate into other myeloid lineage cells (27). Monocytes contribute to innate immunity and inflammation, and high glucose has been shown to enhance toll-like receptor (TLR) expression (28) and upregulate inflammatory genes (29) in monocytes. Notably, the expression of MIP1 in monocytes was shown to depend on OGT, suggesting a link between O-GlcNAcylation and the monocyte’s inflammatory function. The O-GlcNAcylation of E74-like ETS transcription factor 1 (Elf-1) also negatively regulates TOLLIP expression in monocytes (30), which likely enhances monocyte’s responses to TLR activation; however, how this pathway is involved in a real life infection remains to be explored.

Monocytes and macrophages were also shown to play a protective role in cardiac ischemic repair in an O-GlcNAcylation–dependent manner. Zhou *et al.* (31) have convincingly shown that the acute enhancement of O-GlcNAcylation is beneficial and effectively contributes to ischemic repair. It was further shown that the specific O-GlcNAcylation of STAT1 and regulation of myeloid CX3CR1 expression is a mechanism regulating chemotaxis of protective monocytes and macrophages (31). In contrast, the O-GlcNAcylation of STAT3 has been shown to be detrimental in conditions such as inflammatory bowel disease and inflammation induced colon tumorigenesis; its O-GlcNAcylation at T717 inhibits STAT3 Y705 phosphorylation resulting in the loss of IL-10 production in macrophages, leading to exacerbated colon inflammation (32). This shows that even within the same family of proteins and within the same cell type, that is, macrophages, O-GlcNAcylation may impart opposite effects in different tissues or organs.

Monocytes differentiate to macrophages with longer lifespans and continue the innate response. In the initial phase of infection or inflammation, macrophages polarize into a proinflammatory M1 phenotype and during healing or resolution of inflammation they polarize into an anti-inflammatory M2 phenotype (33). Studies on the pathophysiological roles of O-GlcNAcylation in macrophages are relatively larger than in other myeloid cells. Macrophages encountering vesicular stomatitis virus infection display enhanced O-GlcNAcylation and the specific S366 O-GlcNAcylation of mitochondrial antiviral signaling protein (MAVS) and RIG-I–mediated antiviral signaling though activating NF-κB and interferon regulatory factor (IRF)-3 pathways (34). This study also demonstrated that MAVS O-GlcNAcylation is required for and promotes its K63 ubiquitination. Later, it was shown that glucosamine treatment protects mice from multiple lethal virus infections, including influenza A virus (IAV), Coxsackie virus, and vesicular stomatitis virus through antiviral immunity generated through myeloid/macrophage activation of MAVS K63 ubiquitination, IRF3 activation and IFNβ production (35). Another biochemical study on MAVS showed the presence of an O-GlcNAcylation enriched region at amino acids 249–257 and that the sugar modification of this region prevented the dimerization and signaling functions of MAVS. It also blocked MAVS–TRAF3 interaction and interferon production demonstrating O-GlcNAcylation as a negative regulator of MAVS-mediated antiviral immunity (36). This brings up the next set of challenging questions; that is, to distinguish the role of S366 and 249–257 O-GlcNAcylation of MAVS, which likely possess opposing functions, and to define the order, kinetics, and pathological state when O-GlcNAcylation at one or the other site is happening.

Myeloid specific deletion of OGT was shown to cause increased septic inflammation in the mouse model, which was connected with inhibitory O-GlcNAcylation of receptor interacting serine/threonine kinase 3 (RIPK3) at T467 that keeps its phosphorylation under check. It was also shown that O-GlcNAcylation interfered with the function of the receptor interacting protein homotypic interaction motif that mediates RIPK3-RIPK1 heterodimerization and RIPK3 homodimerization. Thus, this study showed two strong biochemical mechanisms involved in macrophage innate immune functions and the suppressive role of O-GlcNAcylation in inflammation (37). Interestingly, RIPK3 was shown to interact with MAVS and also activate the protein kinase R pathway to generate effective antiviral immunity by regulating type 1 interferon production and RIPK3 deficiency was shown to enhance susceptibility to IAV infection. In this case, it appears that RIPK3 checks the activity of the complex-containing MAVS and RIPK1 to achieve necessary IRF3 activation. Since both RIPK3 and MAVS undergo site specific O-GlcNAcylation, it is of interest to know how O-GlcNAcylation is involved in regulating RIPK3–MAVS interaction, and how O-GlcNAcylated RIPK3 is regulating the protein kinase R pathway, to gain an in depth view on O-GlcNAcylation-dependent antiviral innate immune mechanisms. In addition to the upstream regulation of IRF, direct O-GlcNAcylation of IRF5 at S430 was shown to occur following IAV infection, which augments K63 ubiquitination and cytokine production. Adding clinical credibility to this finding, IRF5 O-GlcNAcylation–associated cytokine production was also observed in IAV-infected clinical samples (38).

Apart from regulating upstream interferon pathway proteins, O-GlcNAcylation has been shown to enhance antiviral immunity through the direct regulation of proteins such as sterile alpha motif and histidine/aspartic acid domain–containing protein 1 (SAMHD1). The antiviral mechanisms of SAMHD1 involve the direct degradation of intracellular dNTPs, which limits nucleotide availability for viral replication. The hepatitis B virus infection was shown to cause O-GlcNAcylation of SAMHD1 at S93 and promote its tetramerization and antinucleotide activity (39). Paradoxically, SAMHD1 has also been shown to suppress NF-κB activation (40), inflammatory gene expression, and type 1 interferon activation through MAVS (41). What is the role of SAMHD1 S93 O-GlcNAcylation in these processes emerges as the immediate next question to address.

Apart from viral infections, lipopolysaccharide (LPS) stimulation that mimics bacterial infection was shown to enhance NF-κB p65 O-GlcNAcylation and OGT binding in RAW264.7 macrophages, which was inhibited by glucosamine treatment (42). Consistent with this, an increase in O-GlcNAcylation following glucosamine treatment or OGA inhibition has been shown to offer protection from sepsis in LPS-induced septic shock as well as cecal ligation and puncture models (43, 44). Mechanistically, this anti-inflammatory effect of O-GlcNAcylation was shown to result from the inhibition of NF-κB p65 phosphorylation at S536 (43). However, whether this is a direct competitive inhibition of the S536 phosphorylation site by O-GlcNAcylation or an alternate mode of inhibition is not clear. Although these studies suggest a potential anti-inflammatory role of glucosamine, it is counterintuitive that how glucosamine treatment, which is known to enhance O-GlcNAcylation, is acting as an inhibitor of p65 O-GlcNAcylation, negating LPS-induced O-GlcNAcylation effect, rather than potentiating it. Further adding to the complexity, it has been shown that LPS-induced inflammation is kept under check by a feedback inhibitory loop involving O-GlcNAcylation. LPS transcriptionally activates FoxO1 to induce GFAT2, which enhances cellular O-GlcNAcylation following LPS stimulation, which suppresses NOS2 and proinflammatory cytokine expression (45).

This raises several important questions on the unknowns: what is the need for differential expressions of the two isoforms of HBP rate limiting enzymes, GFAT1 and GFAT2? As macrophages generally express GFAT1, which controls primary O-GlcNAcylation in the cells, what instructs these cells to activate GFAT2 expression under TLR activation? Why this is needed and does this happen in other immune cells as an alternative HBP activation? Does this allude to the possibility that the GFAT1-dependent HBP arm is conserved for normal macrophage functions and that infection triggers an alternative HBP pathway? Also of interest is to learn whether LPS and/or other stimuli alter the expression and function of other enzymes—such as glucosamine-phosphate N-acetyltransferase 1, phosphoglucomutase 3, UDP-N-acetylglucosamine pyrophosphorylase 1, hexokinase, and N-acetylglucosamine kinase—involved in UDP-GlcNAc production in HBP (5).

Microglia is resident macrophages in the central nervous system. Glucosamine was suggested to exert a protective role in neuronal inflammation through correlating its ability to suppress LPS-induced NF-κB activation as assessed by reporter gene assays in the BV2 microglial cell line (42). Similar to NF-κB p65, the NF-κB subunit c-Rel was also shown to bind OGT and undergo O-GlcNAcylation following LPS stimulation, which was antagonized by glucosamine (46). Supporting these studies, it was also shown that the global enhancement of O-GlcNAcylation, following treatment with the small molecule OGA inhibitor, thiamet G, also suppressed inflammation by preventing the polarization of microglial cells from acquiring a proinflammatory phenotype (47). In contrast, LPS stimulation for 30 min was shown to decrease O-GlcNAcylation in N9 microglial cells with a concomitant increase in the phosphorylation of multiple inflammatory mediators (48). Because both of these studies used microglial cell lines, one explanation for the observed differences in the effect of LPS on O-GlcNAcylation could be the kinetics, where LPS transiently suppresses O-GlcNAcylation.

Thinking about the unknowns, it seems possible that although the overall O-GlcNAcylation appears to diminish following LPS stimulation, the O-GlcNAcylation of selected proteins, such as NF-κB c-Rel, may enhance and mediate the proinflammatory functions of LPS. The role of O-GlcNAcylation in other tissue resident macrophages such as splenic macrophages, alveolar macrophages, and Kupffer cells remains unknown and warrants further research to reveal the tissue specific macrophage regulation by O-GlcNAcylation (Table 1).

Unlike macrophages, O-GlcNAcylation remains a largely unexplored area in DCs, which act as bridge linking innate and adaptive immune responses (49). DCs are divided into multiple subtypes, including the three major ones: conventional DC, plasmacytoid DC, and monocyte-derived dendritic cell (moDC) (49). Conventional DCs play major roles in antigen presentation, while plasmacytoid DCs produce type 1 interferon aiding in viral combat, and inflammatory conditions lead to the differentiation of monocytes to moDCs (49). Based on the roles of O-GlcNAcylation in the immune and inflammatory pathways of other immune cells, it is conceivable that the key DC functions such as antigen presentation will also involve O-GlcNAcylation; however, the published experimental evidence in this regard is surprisingly minimal. Several studies have shown the presentation of O-GlcNAcylated peptides by class I major histocompatibility complex (MHC I) in cancer cells, which may contribute to an antitumor T cell response and immune surveillance (50, 51, 52, 53). Whether a similar mechanism exists in MHC I–dependent antigen crosspresentation and MHC II–dependent antigen presentation by DCs is unknown (54, 55).

The inhibition of OGT has been shown to decrease the phosphorylation of AKT in immature DCs, the phosphorylation of AKT, mechanistic target of rapamycin kinase, and ERK in moDCs and the expression of classical DC surface markers and cytokines. It also blocks the transition of monocytes to immature DCs and results in an altered DC phenotype that displays an augmented ability to induce T cell proliferation *in vitro* (19). Although this study provides some leads on the plausible roles of O-GlcNAcylation in DCs, it is necessary to identify subtype and differentiation-dependent changes in O-GlcNAcylation and O-GlcNAcylated proteins in DCs. OGA inhibition, which arrests O-GlcNAc cycling has been shown to cause the differentiation of hematopoietic stem and progenitor cells to DCs; however, the mechanistic details involving O-GlcNAcylation in this process remain murky (18). Independent studies have shown that hyperglycemia enhances O-GlcNAcylation as well as regulates macrophage and DC functions. Macrophages exert deleterious effects in diabetes, through the chronic activation of inflammation and by producing mediators of beta cell death (56, 57) and hyperglycemia have been shown to enhance the inflammatory functions of macrophages (56). An increase in glucose levels also causes DC activation (58). Thus, it is conceivable that hyperglycemia-induced O-GlcNAcylation will have profound roles in skewing macrophage and DC functions under pathological conditions such as type 1 and type 2 diabetes as well as obesity.

Hence, to shed light on these unknowns, discovering the differentially expressed O-GlcNAcylated proteomes in macrophages and DCs under hyperglycemic conditions is necessary. Subsequent functional studies on the newly identified O-GlcNAcylated proteins in the differentiation and functions of M1/M2 macrophages and DCs are expected to further our understanding of how hyperglycemia fuels unmanageable inflammation and unresolved infections occurring in diabetes (Table 1).

## O-GlcNAcylation in NK cell signaling

NK cells are effector lymphocytes that act as innate immune guards, protecting the body from infected and malignant cells. Their activity is controlled through the expression of activating and inhibitory surface receptors, as well as through the secretion of cytokines and cytolytic mediators such as perforin and granzymes (59). Cytokines such as IL-2 and IL-15 are known to activate NK cell function and support their survival (60). Stimulations with IL-2 and IL-15 have been shown to enhance O-GlcNAcylation in primary NK cells along with the expression of the activation marker CD69. Inhibition of O-GlcNAcylation using the OGT inhibitor, OSMI-1, has been shown to decrease the expression of the activating receptors NKG2D and NKp44 and the inhibitory receptor NKG2A and enhance the expression of the inhibitory receptor KIR2DL1, without affecting KLRG1 expression (61). This effect of increasing, decreasing, or not affecting the expression of inhibitory receptors in NK cells suggests that O-GlcNAcylation may regulate the expression of individual genes, rather than a specific function as whole, in NK cells. Apart from the surface markers, blocking O-GlcNAcylation also decreased the cytokines tumor necrosis factor and interferon gamma (IFNG) and the death mediators, FasL, perforin, granzymes, and granulysin and compromised the cancer cell killing potential of both primary NK cells and NK-92 cells (61). In contrast, the coculture of NK-92 cells with cancer cells, which is expected to activate the cytotoxic function of NK cells, has been shown to cause the suppression of global O-GlcNAcylation, along with the enhanced phosphorylation of mitogen-activated protein kinase (MAPK)/ERK kinase and ERK in NK-92 cells (62). This suggests a plausible crosstalk between O-GlcNAcylation and phosphorylation in NK cells.

Directing us toward the unknowns, the discrepancy seen between these studies suggest the possibility of differential regulation of O-GlcNAcylation in NK cells by cytokines and target cell contact, which may play distinct roles in temporal and spatial regulations of NK cell cytotoxic function. Furthermore, the O-GlcNAcylation–dependent changes in cytokine expression and IL-2 responses are suggestive of a role for NF-κB and NFAT pathways in NK cells, which are already well established as O-GlcNAcylation targets in other cell types (63). The immediate questions that demand further research are the following: what specific proteins are O-GlcNAcylated in NK cells in a normal state *versus* a hyperglycemic state? How do viral infections, different tumor types and different tumor microenvironments modulate O-GlcNAcylation–dependent NK cell activity? What are the specific roles of O-GlcNAcylation in circulating and tissue resident NK cells? (Table 1).

## O-GlcNAcylation in mast cell signaling

Mast cells are residents of connective tissue throughout the body and act as key mediators of inflammation following infections or allergic reactions. Trichomonas infection enhances OGT expression and O-GlcNAcylation in mast cells. OGT inhibition has been shown to block reactive oxygen species generation, migration, IL-8 production, and CD63 expression in mast cells treated with Trichomonas secretory products, suggesting a role for O-GlcNAcylation in protozoa-mediated mast cell activation (64).

Into the unknown of mast cells, the first question that arises is how O-GlcNAcylation controls mast cell degranulation that will release factors such as histamine, cytokines, heparin, leukotrienes, and several proteases during allergic responses? Nondegranulated mast cells are necessary for wound healing and diabetes has been shown to accelerate mast cell degranulation, which is attributed as a reason for ulceration and poor wound healing in diabetes patients (65). Whether hyperglycemia also enhances OGT and O-GlcNAcylation in mast cells, similar to protozoan infection, and plays a role in enhanced degranulation is an exciting arena to explore (Table 1).

As a postlude to the discussion on O-GlcNAcylation and innate immunity, one could agree, without hesitation, that the time has passed to study the role of O-GlcNAcylation in other innate immune cells including basophils, eosinophils, NKT cells, and innate lymphoid cells (Table 1).

## O-GlcNAcylation in adaptive immune signaling

Adaptive immunity is the second line of immune response that takes over the tasks initiated and left incomplete by the innate immune response and it creates immunological memory for future immune defense. Adaptive responses are mainly carried out by T and B lymphocytes, whose functions are significantly regulated by O-GlcNAcylation (66).

## O-GlcNAcylation in TCR signaling

Most of the current knowledge on the role of O-GlcNAcylation in the immune system is derived from diligent studies performed using T lymphocytes. Adaptive immunity relies on T cells, which carry out the cellular immune response against infected and malignant cells. Early studies have shown that antigen-independent stimulation of T cells with concanavalin A and phorbol 12-myristate 13-acetate (PMA) results in rapid changes in cellular O-GlcNAcylation suggesting its dynamic role in T cell activation. Cytoplasmic O-GlcNAcylation decreased within 1 h of activation, with a concomitant increase in nuclear O-GlcNAcylation (67), which likely reflects nuclear translocation of O-GlcNAcylated proteins and/or novel O-GlcNAcylation of transcription factors. Consistent with this, pioneering studies have shown that transcription factors such as Elf-1 (68), NF-κB and NFAT (66), c-JUN, JUNB (69), and FOXP3 (15) among several others are O-GlcNAcylated in activated T cells.

Functionally, it was shown that O-GlcNAcylation and PKC theta induced phosphorylation induces Elf-1 nuclear translocation and transcription of the TCR zeta chain, which is compromised in lupus patients, who express lower levels of TCR zeta (68). Similarly, the O-GlcNAcylation of NF-κB and NFATc1 was shown to be necessary for IL-2 transcription in T cells (66), and FOXP3 was stabilized by O-GlcNAcylation (15). O-GlcNAcylation of NF-κB subunit c-Rel at S350 was shown to be required for its CD28RE-dependent DNA-binding activity and for the subsequent activation of IL-2, granulocyte-macrophage colony stimulating factor (GMCSF), and IFNG, yet not for A20 or IκBα expressions, in T cells following TCR stimulation (70). It was also shown that the O-GlcNAcylation of NF-κB c-Rel does not always lead to its activation, as it blocked the DNA binding of c-Rel at the FOXP3 promoter. Thus, it is intriguing to note that O-GlcNAcylation at a single site can have both positive and negative regulatory roles in terms of DNA binding and transcriptional activation. Cocrystallization studies of DNA-bound O-GlcNAcylated NF-κB may prove useful to understand the dual regulatory role of O-GlcNAcylation in NF-κB c-Rel–dependent transcription. Some other unknowns in the biochemical roles of NF-κB O-GlcNAcylation are the following: How does it affects protein–protein interactions and the stability of NF-κB subunits? How does it regulate crosstalk with other PTMs, nuclear translocation of NF-κB, and the duration of transcription? These are key questions which must be addressed in order to delineate the regulation of immune responses through NF-κB O-GlcNAcylation in health and disease.

Both antigen receptor activation and bacterial infection were shown to cause an increase in O-GlcNAcylation as an early event associated with T cell activation, which supports the proliferation and differentiation of T cells. OGT inhibition by genetic or chemical approaches blunts the processes such as the renewal of T cell progenitors, clonal expansion, and malignant transformation (14). Interestingly, c-Myc was shown to be essential for O-GlcNAcylation in activated T cells and c-Myc–deficient T cells failed to increase protein O-GlcNAcylation. This appears to be linked to a role for c-Myc in the expression of glucose and glutamine transporters in T cells (71). TCR activation increases the c-Myc–dependent expression of glucose transporter 1 (GLUT1), a key mediator of glucose transport in lymphocytes (72), which allows additional glucose flux into activated T cells that may enhance cellular O-GlcNAcylation. There exists a feed forward loop where increased glucose flux induced O-GlcNAcylation of c-Myc inhibits its phosphorylation, thereby preventing its degradation, and sustained O-GlcNAcylated c-Myc–dependent facilitation of T cell activation (14). O-GlcNAcylation in T cells was also shown enhanced by Notch signaling that increases glucose and glutamine flux and contributes to β-selection of thymocytes. In line with this, OGT-deficient double negative thymic T cells showed a defective response to Notch activation (14).

O-GlcNAcylation also plays important roles in the generation of different T cell subtypes through the regulation of distinct signaling pathways in T helper cells. It plays a positive regulatory role in augmenting proinflammatory Th17 cells and their function (73). Interestingly, no significant effect of O-GlcNAcylation on Th17 master transcription factor retinoic acid receptor-related orphan receptor γ t variant was observed. However, the enzyme acetyl CoA carboxylase 1, which produces lipid ligands that activate retinoic acid receptor-related orphan receptor γ t variant was activated by its O-GlcNAcylation, likely at multiple sites; S966, S967, S2091, S2285 (73). O-GlcNAcylation exerts two roles in the generation of effector Treg cells and their suppressive function: directly O-GlcNAcylating STAT5 following IL-2 activation, which leads to FOXP3 expression, as well as regulating FOXP3 stability (15).

Some unknowns in the role of O-GlcNAcylation in effector T cell functions are linked with the regulation of STAT5 and STAT1. Because STAT5 and IL-2 have been shown to play a role in IL-4 expression and T helper 2 cell differentiation (74), it is of high interest to know the role of STAT5 O-GlcNAcylation in this process. Like STAT5, STAT1 was shown to be O-GlcNAcylated by inflammatory stimuli that stabilize it in mesenchymal stem cells (MSCs) (75). STAT1 activation in T cells is required for T-bet expression induced by IFNγ (76). Studies on whether O-GlcNAcylation regulates STAT1 phosphorylation and stability in T cells and its role in controlling Th1 differentiation are also warranted.

Antigen recognition by T cells occurs at polarized regions where TCR binds with the peptide presented by the MHC in the antigen-presenting cell. Accumulation of OGT has been observed at the immunological synapse, suggesting that O-GlcNAcylation activity polarizes to the TCR during activation. This may facilitate active substrate modification and the perpetuation of signaling during priming (77), and key proteins binding to the TCR, such as Lck and zeta-chain-associated protein kinase 70, were reported to be O-GlcNAcylated in activated human T cells (77). Providing a big picture view of O-GlcNAcylation in T cells, Woo *et al.* (69) have identified 2219 O-GlcNAcylated peptides from 1045 proteins from activated primary T cells. Similarly, Lund *et al.* (77) came up with 214 O-GlcNAcylated proteins identified from TCR activated primary T cells, and Lopez Aguilar *et al.* (78) identified 445 O-GlcNAcylated proteins in CD8^+^ effector and memory like T cells, over 200 of which were previously unknown substrates of O-GlcNAcylation. Thus, based on these glycoproteomic studies, it is evident that the better part on the role of O-GlcNAcylation in T cells remains unknown and extensive functional characterization of each of the newly identified O-GlcNAcylated proteins in activated T cells is needed to move forward (Table 2).

**Table 2:** Unknowns of O-GlcNAcylation–dependent signaling in adaptive immune cell functions

T cell signaling	B cell signaling
• Mechanisms of positive and negative regulation of transcription factors • Regulation of autoimmunity • Role in T cell sub types: cytotoxic T cells, Th9, Tfh, Th17, Th22 • Functional studies of over 2000 O-GlcNAcylated proteins identified in T cells • Antiviral and antitumor responses • Role in CAR T cells used in cancer therapy	B cell signaling • Transcriptional regulation by NF-κB, NFAT, and other transcription factors • Antibody and autoantibody production and class switching • Antigen presentation • Mature B cell survival and B cell memory • Identification of B cell O-GlcNAc proteome in health, autoimmunity, and malignancy

Representation of major functions of T and B cells that needs further studies to validate or refute the involvement of O-GlcNAcylation.

Abbreviations: CAR, chimeric antigen receptor; NFAT, nuclear factor of activated T cells.

## O-GlcNAcylation in B cell receptor signaling

B cells are involved in humoral immunity through producing antibodies. They also secrete cytokines that contribute to inflammatory pathways and act as antigen-presenting cells activating T cells (79). Knowledge on the regulation of B cell signaling by O-GlcNAcylation is substantially less compared to T cell signaling. B cell receptor (BCR) activation has been shown to cause an increase in O-GlcNAcylation and decrease OGA expression in primary B cells (80). BCR activation increases the expression of GLUT1, possibly through metabolic reprogramming, which may facilitate increased glucose flux, HBP activation, and O-GlcNAcylation in B cells (16, 80). O-GlcNAcylation was shown to be enhanced in rapidly proliferating pre-B cells that consume higher levels of glucose, which was inhibited upon treatment with the OGT inhibitor OSMI-1 (81). OGT inhibition was associated with the downregulation of c-Myc and its downstream genes, cyclin A and cyclin E, in B cells (81). c-Myc is known to be O-GlcNAcylated at T58 (82), which increases its stability, likely supporting pre-B cell proliferation by enhancing c-Myc–dependent cyclin expression (81). Further confirming the role of c-Myc T58 O-GlcNAcylation in B cells, the overexpression of c-Myc T58A was shown to rescue the suppression of pre-B cell proliferation induced by OSMI-1 treatment, indicating a direct role for c-Myc O-GlcNAcylation in B cell proliferation (81).

BCR activation involves the Src family kinase Lyn, which binds to and activates Syk to transduce downstream signaling in B cells. Wu *et al.* showed that BCR activation induces O-GlcNAcylation of Lyn at S19, which was essential for Lyn phosphorylation at Y397 and Syk binding. The substitution of an alanine at S19 was shown to block Lyn O-GlcNAcylation, Syk binding, and abrogated B cell signaling at the receptor level. This is an interesting example of serine O-GlcNAcylation positively regulating the tyrosine phosphorylation of Lyn as well as its protein–protein interaction, projecting O-GlcNAcylated Lyn S19 as a potential target to control BCR signaling (16). Activated B cells also show O-GlcNAcylated lymphocyte-specific protein-1 (Lsp1), which is involved in the antigen receptor mediated apoptotic pathway (83). Lsp1 O-GlcNAcylated at S209 plays a role in the recruitment of PKC-β1, which phosphorylates Lsp1 at S243. This O-GlcNAcylation—phosphorylation crosstalk—leads to the activation of ERK and the subsequent suppression of the antiapoptotic molecules B cell lymphoma-extra large and B cell lymphoma 2, facilitating apoptosis (80). Escape from BCR-mediated apoptosis relies upon B cells securing other survival signals such as CD40 or B cell–activating factor receptor (BAFF-R) activation, both of which have been shown to activate key transcription factors, such as NF-κB and NFAT that regulate survival pathways. BCR activation and high glucose increases the O-GlcNAcylation of NF-κB and NFATc1 (66) as part of regulating B cell function. Intriguingly, NFATc1 was shown to be rapidly O-GlcNAcylated at 5 min following BCR activation, suggesting a dynamic role of O-GlcNAcylation in BCR signaling. Further direct functional studies are necessary to show the specific roles of O-GlcNAcylated NF-κB and NFATc1 in B cells.

The conditional deletion of OGT using CD19-Cre that results in gene knockout starting pre B cell stage was shown to cause increased apoptosis of mature B cells, indicating the critical role of O-GlcNAcylation in B cell survival. Interestingly, the number of immature B cells was found unaffected by OGT deletion, indicating enhanced cell death in mature B cell populations. This was attributed to compromised survival signaling emanating from the B cell–activating factor receptor (16). The majority of the mature B cell apoptosis happened in the pool of follicular B cells in the CD19-Cre/OGT KO mice, while other B cell compartments were minimally affected. Because the CD19-Cre deletion of OGT did not show any significant effect on marginal zone B cell death due to unknown reasons, further studies were conducted following the specific deletion of OGT in marginal zone B cell using IghCγ1-Cre mice. This comprehensive study demonstrated that O-GlcNAcylation is also critical in regulating the survival of marginal zone B cells and memory B cells as well as antibody production by B cells/plasma cells (16). The details of O-GlcNAcylated proteins and their specific roles in follicular and marginal zone B cells, memory B cells, and plasma cells remain unknown. How O-GlcNAcylation regulates cytokine production and antigen presentation by B cells and how these functions are altered under hyperglycemia also remains unknown. BCR activation has been shown to induce O-GlcNAcylation–dependent changes in the phosphorylation of over 300 proteins in B cells in a proteomic study (80). This tells us that with the handful of O-GlcNAcylated proteins that have been studied in B cells so far, we are just at the doorstep and there is much more unknown information that must be explored in order to understand the role of O-GlcNAcylation in B cell signaling.

Overall, deciphering discrete mechanisms regulated by O-GlcNAcylation in adaptive immunity may provide new avenues for modulating immune responses in various infections, autoimmunity, cancer, and cancer immunotherapy and for improvising vaccine development (Table 2).

## O-GlcNAcylation and immunometabolism

Cellular metabolism is fundamental in providing the energy and building blocks for cells in homeostasis. It also provides the need based enhanced energy supply for the immune system at times of immune activation (84). In addition to energy production, a large number of recent studies have revealed the role of immunometabolism in regulating the immune response through the modulation of immune cell signaling, which is still an emerging area of research (85) where the known likely remains infinitesimal compared to the unknown. Among all the metabolic pathways regulating immune cell signaling, HBP and O-GlcNAcylation remains unique in that it has a direct effect on signal transduction as a PTM that influences other PTMs, mainly phosphorylation as well as ubiquitination, acetylation, and methylation. Interestingly, GLUTs and several proteins regulating glycolysis, such as hexokinase, phosphofructokinase, and pyruvate kinase, are also substrates of HBP (86). Most of the enzymes in the tricarboxylic acid cycle were also shown to be O-GlcNAcylated (87). Current knowledge on the role of O-GlcNAcylation in immunometabolism is mostly derived from studies performed in T cells (88). Both glucose and glutamine are essential for T cell activation and HBP. O-GlcNAcylation dynamically changes during stages of T cell development and it regulates thymocyte metabolism by regulating Notch and c-Myc functions (14). O-GlcNAcylation was also shown to be essential for lineage stability and T cell suppressive function in Treg cells. TCR-induced O-GlcNAcylation was shown to stabilize FOXP3 and activate STAT5 (15), as well as regulate insulin sensitivity and iron metabolism in Treg cells in a STAT5-dependent manner (89). Like Treg cells, MSC are also involved in suppressing T cells to maintain homeostasis. Inflammatory conditions result in enhanced STAT1 O-GlcNAcylation in MSC that was shown to promote its T cell suppressive function (75). Similar to T cells, O-GlcNAcylated c-Myc has also been shown to play key roles in glucose uptake and B cell proliferation (81). In macrophages, O-GlcNAcylation has been shown to be a homeostatic regulator of metabolism and polarization through controlling S6 kinase beta-1/mammalian or mechanistic target of rapamycin kinase complex 1 signaling (90). Thus, it appears that O-GlcNAcylation substantially affects metabolism-dependent functions of T cells, B cells, and macrophages.

## Unknowns in O-GlcNAcylation and immunometabolism

**Figure 1:**
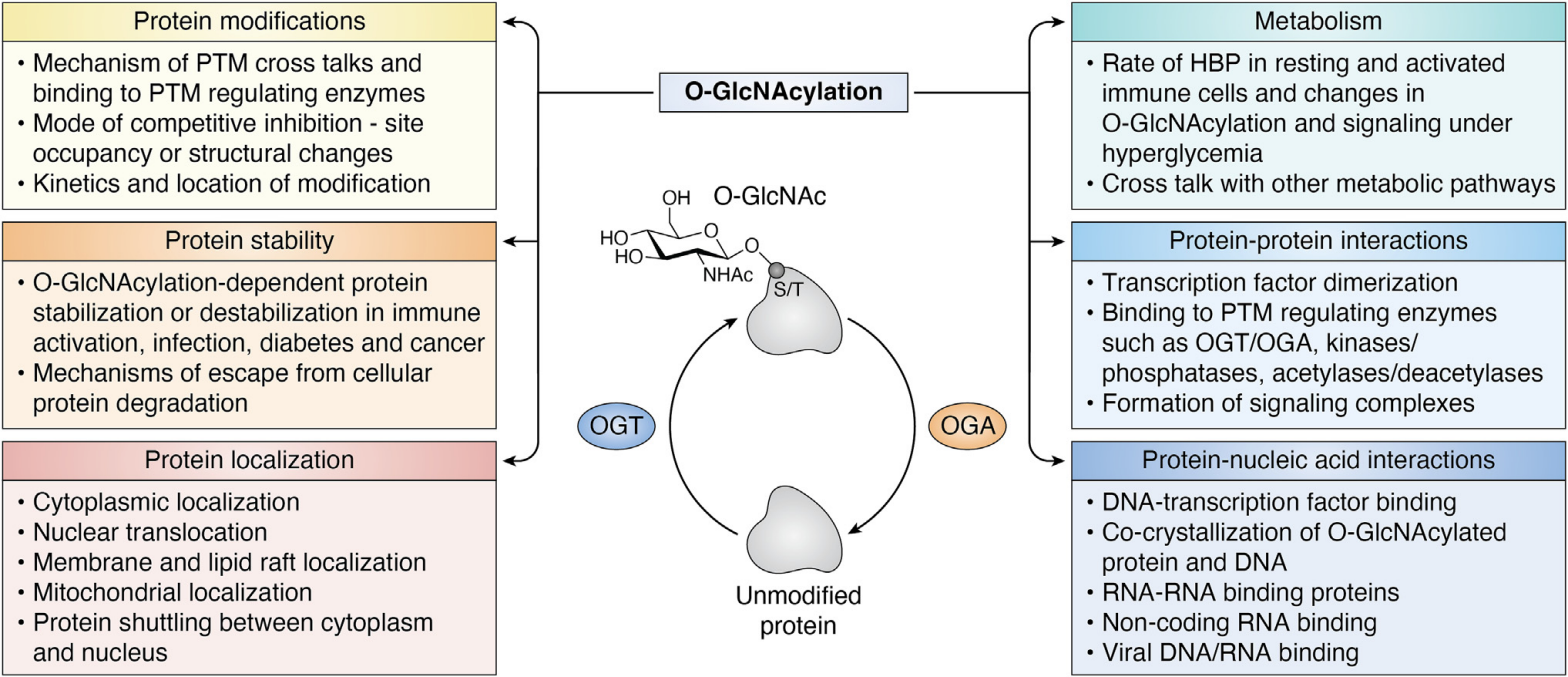
**Unknowns in the biochemistry of O-GlcNAcylation–dependent signaling.** Representation of key biochemical events in protein and nucleic acid signaling that are likely regulated by O-GlcNAcylation at global and protein specific levels.

Overall, the relationship between the availability/utilization of glucose and HBP has not been established well in immune cells or in healthy *versus* disease conditions. Several studies approximated a diversion of about 2 to 5% of glucose entering the cells to HBP, based on seminal work done on adipocytes (91). However, a comprehensive study conducted using mouse heart demonstrated a huge discrepancy in the widely acknowledged share of 2 to 5% of glucose entering HBP and showed that only ∼0.003 to 0.006% of glucose is channeled through HBP (92). This finding raises several questions: is HBP rate cell type–dependent and, if so, what is its percentage in immune cells? Does it correlate with the energy requirement or glucose consumption of the cell? Does a high-energy requiring cell type, such as heart, favor glucose input into other metabolic pathways and lower HBP? What are the rates of HBP in resting and activated immune cells? How does the chronicity of inflammation or infection affect HBP and O-GlcNAcylation-dependent signaling in immune cells? What is the actual variation of HBP rate in hyperglycemic conditions such as diabetes and obesity? Are the expressions of OGT and OGA or the rate-limiting enzyme GFAT, regulated to fine-tune HBP in health and disease? Is there a correlation between HBP and other metabolic pathways including the pentose phosphate pathway, oxidative phosphorylation, and the polyamine amine biosynthesis pathway that control immune function? Thus, opening the treasure chest of O-GlcNAcylation and immunometabolism is expected to take us leaps forward with a wealth of knowledge in the world of biochemistry of the immune system (Fig. 1).

## Uncommon biochemical approaches to tackle unknowns in O-GlcNAcylation

In general, it appears that proinflammatory or anti-inflammatory effects of O-GlcNAcylation are cell type– or pathology-dependent. Because glucosamine has been shown to cause global elevation of O-GlcNAcylation (93), several challenging questions emerge such as how glucosamine stimulation and associated increase in O-GlcNAcylation can have reciprocal regulatory roles in the cells? How is the dosage of O-GlcNAcylation of individual proteins adjusted? How does the crosstalk of O-GlcNAcylation with phosphorylation at a global level happen to have a proinflammatory or anti-inflammatory outcome? One approach to tackle this issue is to develop highly sensitive approaches to simultaneously detect proteins with O-GlcNAcylation and phosphorylation, as well as other PTMs, and quantitate their extent of modifications in physiological and pathological conditions. The field has started venturing on discovering such approaches as seen in multiple previous studies. Multiplexed approaches that have been developed to detect O-GlcNAcylation and phosphorylation in gel appear promising; however, they come with the limitation that they are only suitable only for abundantly expressed proteins (94). A mass spectrometry–based study has shown that O-GlcNAcylation predominantly happens on the same proteins that are phosphorylated (95), justifying the potential role of their crosstalk in biology. A recent technique combining metabolic labeling, hydrophilic interaction liquid chromatography, and mass spectrometry has shown success in identifying O-GlcNAcylation and phosphorylation in RNA-binding proteins (96), offering a technique that may be improvised for other proteins as well. Another recent study took an additional step and systematically analyzed O-GlcNAcylated and phosphorylated proteins in the cytoplasm and nucleus and showed that both modifications are enriched in the nuclear fraction (97), possibly due to the contribution of PTMs to the nuclear transport of proteins. Thus, comprehensive biochemical studies seem to be the right approach to apply to learn discrete mechanistic roles of protein O-GlcNAcylation in controlling various immune cell functions. Significant efforts are also needed to develop innovative biochemical approaches to study the role of O-GlcNAcylation in protein–protein and protein–DNA/RNA interactions, which ensures the maintenance of O-GlcNAcylation of the proteins in the examined complexes. Such studies come with the dual benefits of deciphering novel mechanisms involving O-GlcNAcylation and revealing potential therapeutic targets (Fig. 1).

## Crosstalk between O-GlcNAcylation and other PTMs

**Figure 2:**
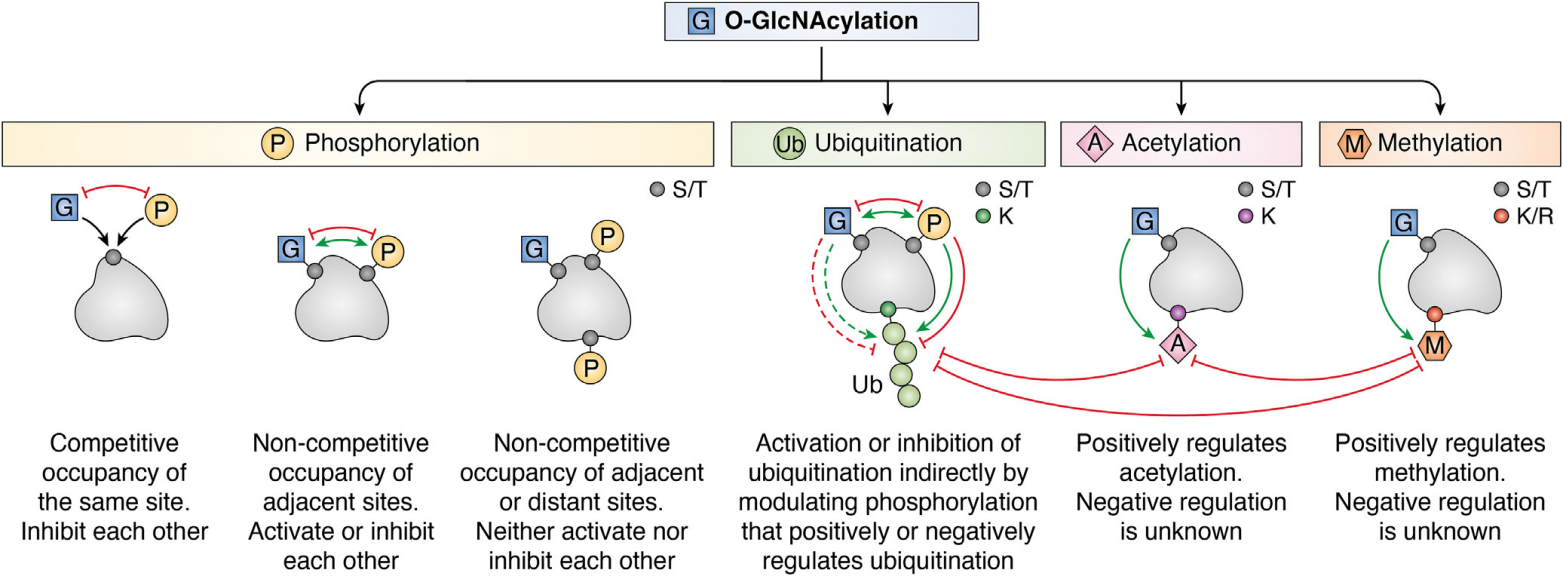
**Modes of crosstalk between O-GlcNAcylation and other PTMs.** S-serine, T-threonine, K-lysine, R-arginine. *Red lines* indicate inhibition and *green arrows* indicate activation. Potential indirect effect of O-GlcNAcylation on ubiquitination is shown using *dashed lines*. Possible competition or cross inhibition of the same lysine residue by methylation, ubiquitination, and acetylation are also shown. PTM, posttranslational modification.

A quick look into the literature garners attention to the crosstalk between O-GlcNAcylation and phosphorylation that suggests three modes of coexistence of these PTMs based on their proximity (4, 9, 96, 97, 98). First, both PTMs target serine and threonine residues, resulting in the competitive occupancy of the same site, in which one modification blocks the other. Second, a noncompetitive occupancy of adjacent sites, where one modification may either facilitates or inhibits the other modification. Third, a cooperative occupancy, where O-GlcNAcylation and phosphorylation happens in adjacent or distant sites and they may neither activate nor inhibit each other (Fig. 2). It would be interesting to study the function of an O-GlcNAcylated phospho-protein in a pathophysiological condition to examine how simultaneous dual PTMs regulate a protein’s function. In the case of competition between O-GlcNAcylation and phosphorylation for the same site, it remains largely unknown whether the blockage of one modification by the other happens through simple site occupancy or if it affects binding to the OGT/OGA or the respective kinase, through structural changes.

In addition to phosphorylation, there exists ample evidence for the link between O-GlcNAcylation and other PTMs, such as ubiquitination (99), acetylation (100), and methylation (101) (Fig. 2). O-GlcNAcylation may interfere with ubiquitination by directly competing with the phosphorylation sites that prime the proteins for ubiquitination and degradation and by facilitating the binding of proteins with deubiquitylases. It is also possible that O-GlcNAcylation may enhance phosphorylation that inhibits ubiquitination (102). The predominant outcome of this crosstalk is the stabilization of the O-GlcNAcylated protein through interference with the ubiquitination/degradation pathway, although some exceptions exist, such as the destabilization of O-GlcNAcylated A20 under inflammatory conditions (103). Such enhanced protein degradation might result from possible noncompetitive O-GlcNAcylation sites facilitating phosphorylation that primes ubiquitination, a mechanism that remains to be demonstrated through further research (Fig. 2). Crosstalk between O-GlcNAcylation and ubiquitination has been discussed in detail previously (99).

Limited evidence is available on the crosstalk of O-GlcNAcylation with acetylation and methylation. NF-κB p65 O-GlcNAcylation at T305 has been shown to potentiate its acetylation at K310 by p300 in nonimmune cells, which regulate cell survival signaling (100). It is highly likely that a similar regulation of NF-κB exists in immune cells as well and learning its immune function in physiological and pathological contexts is of high interest. It is noteworthy that O-GlcNAcylation and acetylation are dependent on glucose metabolism that generates their intermediate substrates, UDP-GlcNAc, and acetyl CoA, respectively. Hence, the nutrient availability and metabolic status of the cells are expected to have a significant impact on these PTMs and associated cellular functions.

The overexpression of OGT enhances K9 acetylation and K27 trimethylation of histone 3 that plays a role in mitosis (104). OGT was also shown to bind to the ten-eleven translocation proteins TET2 and TET3 and O-GlcNAcylate host cell factor-1, a shared subunit of the H3K4 methyltransferase Su(var)3-9, Eenhancer of zeste [E(z)], and trx-1 (SET1)/complex of proteins associated with SET1 (COMPASS) complex, which allows the sustained K4 methylation of histone 3 (105). The TET family of proteins was shown to play critical roles in CD4^+^ T cell differentiation (106) and stabilize FOXP3 expression in Treg cells (107). They also regulate CD8^+^ T cell functions, and the loss of TET2 has been shown to promote the differentiation of CD8^+^ memory T cells (108). These findings suggest the potential role of O-GlcNAcylation in epigenetic regulation, through its effects on acetylation and methylation (109).

Furthermore, O-GlcNAcylation has also been shown to influence other PTMs, such as poly-ADP-ribosylation (PARylation) (110) and S-nitrosylation (111). O-GlcNAcylation of the enzyme poly ADP-ribose glycohydrolase, which removes PARylation, enhances its nuclear localization and recruitment to DNA damage sites. This in turn facilitates the PARylation of cytoplasmic DNA damage–binding protein 1 and its stabilization, by diminishing its autoubiquitination (110). This study reveals an interesting crosstalk involving O-GlcNAcylation, PARylation, and ubiquitination, which may be applicable to other proteins as well. Adding further to the list of crosstalks, a reciprocal relationship between O-GlcNAcylation and S-nitrosylation has been shown, in which S-denitrosylation of OGT results in its enhanced activity under heat shock stress (111). Overall, ample evidences exist to show that O-GlcNAcylation crosstalks with most of the other PTMs, leading to the altered stability, activity, and localization of various proteins. As discussed above, three things are needed to advance the field: the biochemical approaches to simultaneously study O-GlcNAcylation with other PTMs, the identification of modification sites, and site-directed mutagenesis studies in relevant cell types to elucidate the function of specific O-GlcNAcylation (Figs. 1 and 2). In addition to the protein–protein interaction studies, it is essential to conduct in depth investigations on how crosstalk between O-GlcNAcylation and other PTMs mechanistically regulate protein–DNA interactions and transcription. Considering that a large number of O-GlcNAcylated proteins identified to date are involved in transcription, it is necessary to study how O-GlcNAcylation directly or indirectly affects other PTMs in the same or interacting proteins to temporally and spatially control transcription. This should be followed up with *in vivo* studies in animal models and, depending on the context, validation experiments should be performed using relevant patient samples.

## O-GlcNAcylation as a potential therapeutic target for immune cell diseases

There are a growing number of autoimmune and inflammatory diseases, as well as cancers, where O-GlcNAcylation and its dysregulation have been implicated (6, 112, 113, 114). Autoimmune diseases including lupus, rheumatoid arthritis (RA), and type 1 diabetes show dysregulated O-GlcNAcylation. An increase in OGT expression has been reported in the CD4^+^ T cells of lupus patients (115), which may increase global O-GlcNAcylation, altering immune cell signaling resulting in an overactive immune system and inflammation. In contrast, it was also reported in lupus patients that Elf-1 O-GlcNAcylation–dependent TCR zeta expression was downregulated (68), which suggests a lack of direct correlation between the mere increase in O-GlcNAcylation and lupus pathology (Table 3).

**Table 3:** Unknowns of O-GlcNAcylation–dependent signaling in immune diseases

Disease	Challenges
Type 1 diabetes, Type 2 diabetes, and obesity-associated hyperglycemia	Hyperglycemia increases global O-GlcNAcylation levels. Specific changes associated with enhanced O-GlcNAcylation and disease-specific altered protein function compared to healthy counterparts is largely unknown.
Secondary complications of diabetes	How chronic increase in blood sugar and associated O-GlcNAcylation affects multiorgan inflammation in diabetic retinopathy, neuropathy, and nephropathy as well as cardiovascular complications?
Chronic inflammation (*e.g.*, inflammatory bowel disease, rheumatoid arthritis)	How O-GlcNAcylation contributes to sustained inflammation or prevents resolution of inflammation in conditions such as inflammatory bowel disease and rheumatoid arthritis?
Autoimmunity (*e.g.*, type 1 diabetes, lupus)	How O-GlcNAcylation controls the autoreactivity of immune cells such as cytotoxic T cells and autoantibody-producing B cells in autoimmune diseases such as type 1 diabetes and lupus?
Cancer (*e.g.*, chronic lymphocytic leukemia, acute myeloid leukemia)	Identification of key O-GlcNAcylated proteins and the specific site(s) of modification that contribute to the cause, progression, and relapse of cancer
OGT/OGA SNPs in disease	Identification of SNPs in OGT or OGA specifically associated with a disease that may result in increased or decreased O-GlcNAcylation

Representation of selected autoimmune, inflammatory, and malignant diseases where O-GlcNAcylation is implicated and immediate challenges that needs further studies to validate or refute the translation potential of O-GlcNAcylation.

Abbreviations: OGA, O-GlcNAcase; OGT, O-GlcNAc transferase.

In RA, inflammatory stimuli, such as tumor necrosis factor and receptor activator of nuclear factor kappa B ligand, have been shown to cause the O-GlcNAcylation of nucleoporin 153 at T546 and S548. It enhances nuclear Myc activation and promotes osteoclastogenic gene expression, and blocking OGT has been shown to offer benefits in preventing bone loss in animal models (116). The anti-inflammatory molecule penta-O-galloyl-beta-D-glucose has been shown to block transforming growth factor-beta activated kinase 1-binding protien 1 O-GlcNAcylation and decrease its binding with TAK1, blocking IL-1–induced inflammation in RA (117). Thus, these studies suggest that O-GlcNAcylation exerts a pathogenic role in RA (Table 3).

In type 1 diabetes, O-GlcNAcylation appears to play a dual, but reciprocal, role through modifying the NF-κB c-Rel protein at S350. The O-GlcNAcylation of c-Rel at S350 results in its enhanced binding at the promoters of IL-2, granulocyte-macrophage colony stimulating factor, and IFNG (70), and decreased binding at the FOXP3 promoter (118), the combined outcome of which might be the exacerbation of autoimmunity (Table 3).

## Into the unknowns of translational potential of targeting O-GlcNAcylation

Targeting O-GlcNAcylation, though sounds luring, is something, which must be considered with a deep understanding of the condition, where its effect is leading to a pathological consequence. Any therapy has to be formulated in a way that does not affect the vital basal levels and functions of O-GlcNAcylation. Hence, on a first look, inhibiting O-GlcNAcylation appears to be an option to control diabetes and cancer, because both of these conditions show significantly enhanced cellular O-GlcNAcylation (5). This would call for the optimization of a therapeutic dose of the drug specifically to diminish consequences of enhanced O-GlcNAcylation. Because OGT is critical for survival (2), while inhibiting OGT to block O-GlcNAcylation, it is important to know what is the tipping point for the cell before they succumb to death. On the other hand, Alzheimer’s disease seems to respond well to treatment that enhances cellular O-GlcNAcylation through OGA inhibition (119). Here, the observed favorable effect occurs through the arrest of O-GlcNAc cycling, rather than an absolute enhancement of HBP and rate of O-GlcNAcylation, which raises an interesting question on the long-term consequences of OGA inhibition. This is significant because the knockout of OGA has been shown to cause early lethality (3), which suggests that similar to its addition, removal of O-GlcNAcylation is also equally important for homeostasis.

Cancer cells, including lymphoid and myeloid malignancies, display enhanced levels of O-GlcNAcylation, likely due to their relatively higher levels of glucose flux and altered growth kinetics (112, 113). Cancer cells also show increased OGT expression, which increases O-GlcNAcylation and promotes cancer cell proliferation and resistance to chemotherapy (120). Based on this, OGT and O-GlcNAcylation inhibition have been proposed as potential therapeutic approaches for the treatment of cancer (114). On the other hand, O-GlcNAcylation also controls the activation, survival, and function of T cells and other immune cells (4), which are critical in fighting cancer. This demands cancer cell specific suppression of OGT through optimizing the dose range of OGT inhibitors and/or genetic targeting of OGT in cancer cells. Modulating O-GlcNAcylation in the immune cells to enhance their natural healing capabilities and fight against cancer cells also holds the potential to lead toward improved cancer treatment outcomes (Table 3).

In line with the above-mentioned roles of O-GlcNAcylation in regulating viral infections, recent pandemic related studies have also linked it to severe acute respiratory syndrome-related coronavirus-2 infections. Diabetes and hyperglycemia have been linked to more severe CoV-2 infections (121), suggesting a possible role of O-GlcNAcylation in the hyper cytokine storm response reported in diabetes patients. A thoughtful working hypothesis was published on the potential role of O-GlcNAcylation in CoV-2 infection, extrapolating the knowledge gained from IAV infection. It was proposed that CoV-2 infection would enhance glucose consumption, OGT expression, and O-GlcNAcylation, to favor the viral replication and activation of interferon pathways leading to NF-κB activation and hyper inflammation (122). Although not supported by experimental evidence, this hypothesis appears to depict real life situations, as it aligns well with the available information discussed above on the regulation of viral infections by O-GlcNAcylation.

## Conclusion and future perspectives

Because O-GlcNAcylation has the potential to significantly alter immune responses, understanding the precise molecular mechanisms involving O-GlcNAcylation in the immune system is critical for developing new therapeutics for immune and inflammatory diseases, as well as for lymphoid and myeloid cancers. Further research is needed to understand the intricacies of targeting OGT and OGA, as these are unique enzymes controlling a plethora of vital functions. OGT and OGA are large proteins with molecular weights of over 100 kDa and the SNP database shows that several mutations exist throughout their sequences. However, functional significance and pathophysiological consequences of these variants remain unknown and warrant further research. As novel techniques are continuously developed to study O-GlcNAcylation and high throughput and single cell studies are expanding, along with artificial intelligence and robotics, there is a large scope to advance research on the role of O-GlcNAcylation in the immune system. The goals should be to utilize the growing knowledge to aid in developing strategies to target disease-specific increased or decreased global O-GlcNAcylation, as well as to target site-specific O-GlcNAcylation of proteins to yield novel therapeutic approaches. Such site-specific and protein-specific O-GlcNAcylation targeting reagents and approaches, which will most likely emerge from biochemical studies on protein–protein and protein–nucleic acid interactions, protein stability and localization, as well as the interface of metabolism and signaling (Fig. 1), are expected to yield therapeutics with minimal off target effects. O-GlcNAcylation has been shown to regulate genes involved in self-renewal and pluripotency in stem cells (123), which control the differentiation of stem cells to other cell types in the body. Thus, in addition to potential chemical and genetic therapeutics targeting O-GlcNAcylation, targeting O-GlcNAcylation and O-GlcNAcylated proteins in stem cells allow their manipulation to accelerate cell therapy based therapeutic development in regenerative medicine as well.

## Conflict of interest

P. R. has two patents encompassing O-GlcNAcylation in the immune system, US09696296B2 and US20220259275A1.
